# Case of Hydrophobia

**Published:** 1815-09

**Authors:** G. Pinckard

**Affiliations:** Bloomsbury-Square


THE LONDON

### Medical and Physical Journal.

3 OF VOL. XXXIV.] SEPTEMBER, 1815. [NO. 199.

"For many fortunate discoveries in medicine, and for the detection of numerous errors, the world is indebted to the rapid circulation of Monthly Journals; and there never existed any work to which the faculty in EUROPE and AMERICA were under deeper obligations than to the Medical and Physical Journal of London, now forming a long, but an invaluable, series."—RUSH.

*For the London Medical and Physical Journal.*

### *Case of Hydrophobia*; by Dr. PINCKARD.*

JOHN TIFF, a gardener, ætat. 34, a healthy strong man, residing near the Toll-gate at Kilburn, Middlesex, was working in a field at the back of his house early on the morning of Monday, May 8, 1815, when a large Newfoundland dog, running down from the public road, bit him severely in the left leg, tearing the flesh, so as to cause several extensive wounds.

He went immediately into the house and sent for Mr. Rogers, a surgeon residing in the place, who was with him in less than half an hour after the accident. The wounds were washed with hot vinegar, repeating it twice each day, and the man was put upon a course of the Ormskirk medicine, which was taken internally, and applied to the part regularly for five weeks according to the printed instructions of the proprietor.

During this time he remained well and the wounds healed kindly; one only remaining open, which was likewise in a healing state.

On Tuesday, June 13th, thirty-six days after the time when he was bitten, the wounded part of the leg was attacked with severe pain, which proceeded up the thigh, affected the inguinal glands in such a degree as to cause a tenderness or soreness, and extended itself acutely across the back.

Not suspecting the nature of this seizure, he called it rheumatism, and, mentioning it to a neighbour, who chanced to come into the house, his friend rubbed his limb and his back with some oils, which he had himself been accustomed

* This paper was sent us for insertion last month, but long after the department allotted for Original Communications was closed. We must again request our friends, who wish that their communications should appear early, to favour us by sending them early in the month.—EDIT.

to use for rheumatic pains. During this application, a drop of the oil happened to fall upon his thigh, when he was instantly seized with a sense of suffocation, and breathed with a spasmodic catching, like that which a person feels upon descending suddenly into cold water.

The pain not being removed, he sent for Mr. Rogers, who saw him about eleven o'clock in the evening, and gave him a draught with forty drops of the tincture of opium. He passed a disturbed and most wretched night, being unable to sleep or to lie still, and suffering very severely from pain, and excessive irritability. The feeling of suffocation also increased to a distressing degree, and was accompanied by spasmodic startings and flatulent eructations.

In the morning of June 14th, all the characteristic symptoms of that most melancholy and fatal disorder, hydrophobia, were present. The moving of water in the room, fluids being brought within his sight, or any thing being presented for him to swallow, threw him into violent agitations, amounting almost to convulsions. The same effect was produced also by disturbing the air of the chamber, opening the door or the window, breathing upon his skin, lifting up the bed clothes, using a pocket handkerchief near him, or to wipe his face, or even by a fly settling upon his countenance, or a drop of perspiration falling from his brow upon any part of his person.

Great restlessness and anxiety, a dread of suffocation, and a general sense of terror or apprehension prevailed, while sleep and quietude seemed to be utterly banished.

About the middle of the day on the 14th, a medical attendant from London administered to the poor man four bottles of vital air, and directed two bottles of it to be repeated at night and early the next morning. He prescribed likewise two grains and a half of the nitrate of silver to be taken every four hours, and ten drops of muriatic acid during the intervals between each dose.

The night of the 14th was passed in the same afflicting manner as that preceding; but the symptoms were more aggravated, and the sufferings, if possible, more extreme. All the senses seemed to be rendered painfully acute, and the susceptibility of impression was vivid and distressing beyond description. Added to the means before related, he was now greatly hurried and almost thrown into convulsions by the shutting of the chamber door, the snuffing of the candle or any other sound, and he remarked that even closing his eyes produced great agitation and spasmodic startings.

On the morning of Thursday, June 15th, Mr. Smith, surgeon, of Greville-street, who very humanely paid great attention to the afflicted sufferer, requested me to visit him.

On

On my arrival, about twelve o'clock, I found him sitting up in bed, distressed with almost constant eructations, dry retching, and a sense of suffocation, which prevented him from swallowing and from lying down. He was collected and rational; but his eyes were widely opened, giving rather a staring look to his countenance, which was otherwise natural. He seemed watchful and apprehensive; and his manner was expressive of great anxiety and disquietude. He felt a sort of comfort and protection from having people about him, particularly medical men. When Mr. Smith and myself entered his room, he looked consoled, and expressed himself thankful. A heavy perspiration was diffused over his person, and stood in large drops upon his face. He was still agitated, and breathed with spasmodic catchings when any fluid was presented to him to drink, or when the air was moved about his person, but in a far less degree than he had been on the preceding day. He swallowed his saliva, and had no longer a dread of seeing water, but took a glassful into his hand, in which were a few drops of the muriatic acid, saying, "I could drink it in a minute if I could break off the wind, which is choaking me;" and adding, "I have had a hard struggle for it, but have overcome it at last, and can now swallow: I can both eat and drink to-day better than I could yesterday." Still he put down the glass, and took it up, several times before he could carry it to his mouth; and, when it reached his lips, repeated eructations and startings prevented him from swallowing the fluid; but he was very resolute and determined, and, after persevering for nearly a quarter of an hour, he accomplished the task by drinking the contents of the glass in a hurried and convulsive manner, first desiring the window to be closed, that the air might not blow upon him, and, stopping, when he had taken about the half, saying, "I can finish it when I have got rid of the wind," which he did, but with an effort which threw him into increased perspiration, and produced flatulent and convulsive retchings.

On my opening the window again afterwards, he remarked that the air "choaked" him, and desired to have the sash put down. He complained of thirst, but observed that he had no sense of heat, or soreness at the stomach, nor did he express any pain or tenderness upon pressure either of the stomach or any part of the abdomen, although there was considerable tension of the latter, and he constantly placed his hand over the former, observing, "If that were removed, I should be well; but if you can't move that, it will kill me"—meaning the uneasy sensation from distension and flatulency.

 Occasionally

Occasionally the eructations were accompanied with a degree of suffocation and starting, which caused him to draw himself up, and throw back his person, crying out convulsively, "oh dear! oh dear!"

The skin was moderately cool; the tongue moist and clean; the bowels had been opened on the 13th; the pulse was nearly 120, not full nor hard, but slightly tense.

Forty ounces of blood were directed to be taken from the arm. In preparing for the operation, a person seated himself at his back, in order that he might be supported by leaning against him, when, from the man happening to breathe upon him, he started convulsively forward, calling him by name, and crying out impatiently, "Why do you blow upon my neck!"

Mr. Rogers opened a large orifice in the vein, and the blood flowed quickly; but the colour did not leave his countenance, nor did he become faint. He looked more composed and tranquil, appeared less anxious, and was able to wipe the perspiration from his face with a handkerchief during the bleeding. On being asked if he felt better, he said, "I must give some consideration before I answer that;" and presently he remarked, "I feel more easy and comfortable—the bleeding takes away the stuffing at my stomach." Profuse perspiration continued. The pulse became more expanded, and was rather diminished in frequency; but, upon the whole, the relief from drawing blood did not seem to be greater than might proceed from his attention being diverted by the circumstances of the operation. The blood was of natural appearance.

After the vein was closed, I asked him to try if he could lie down; but he begged to "sit a-while first;" and presently, upon attempting to recline, the startings, eructations, and spasmodic breathing, became violent, and he leaned back, looking exceedingly distressed, and gulping, as if trying to swallow, crying out several times, "oh dear! oh dear! if I could but get that up! Oh dear! Lord have mercy upon me! I must go if I don't get rid of that"—still placing his hand over his stomach, and referring to the oppressive and suffocating sensation which so cruelly afflicted him.

He seemed timid of being left without some one close by him; and, upon the man who was sitting at his side happening to rise from the chair, he instantly became hurried and agitated, and intreated him not to go away, saying, "When it comes on, I can't govern myself."

He now desired that nothing more might be given to him until the "stuffing" was removed, and, pressing upon his stomach, stomach, said, "It is here, and I feel my throat very dry: if I try to take the powder, I shall be choked: O, if I could but get that off, I should be well." "It will serve me so again presently;" meaning that the spasmodic starting would return.

Notwithstanding the bleeding, and all the means used, the symptoms of the disease were rapidly proceeding in their usual course, the alleviation was but momentary, and the diminished agitation from feeling the air, or from the approach of liquids, only proved the disorder to be advancing to its last stage.

Mr. Smith and myself remained with him until it was past three o'clock. At this time he asked for drink, and desired to have cold water, of which he swallowed a wine-glassful in a hurried manner, but with far less agitation and difficulty than he had taken the water, with the drops, only a short time before. He also allowed the window at the side of his bed to be opened without complaining of the air, although it blew directly upon his person. He still said, "All I feel is here," placing his hand, as before, upon the region of his stomach.

On our moving to leave the room, he seemed distressed, and inquired anxiously if we should soon return, or if "the other doctors" would come to him presently. The nitrate of silver was continued; but the spasms and convulsive startings increased, and the eructations and retchings became almost incessant. At intervals he called out, in great agitation, "Take care! I feel the fit coming on—oh dear! oh dear!" His limbs gradually became colder and colder; he grew weaker; his pulse was quick and feeble; and, from not being able to swallow the saliva, it collected about the corners of the mouth, where, from his breathing through it, it formed a frothy appearance.

Feeling his strength decline, he was sensible of his approaching dissolution, and, looking anxiously round to his friends who were in the room, he called them by their several names, remarking to them that his was an awful death, and imploring them to comfort and protect the dear partner of his days, whom he was about to leave a widow; then, desiring to see his wife and children, he took leave of them in the most impressive and affecting manner, tenderly bidding them farewell, and reminding them, with the sublime resignation and fortitude of a Christian, that they would meet again in a happier world. His extremities were now cold and livid, but he continued to hold the hand of his poor weeping wife, affectionately pressing it whilst life remained.

He was sensible to the last; and, calmly sinking, as it were, into into a tranquil sleep, he expired at a quarter before twelve o'clock on Thursday night.

The duration of the disease was about fifty-eight hours from the time when the pain of the leg was first perceived. In its course it exhibited several periods or stages, which were marked with tolerable accuracy, and may be briefly stated under the following heads:

1st. Acute pain, extending from the bitten part, about fourteen hours.

2d. A dread of liquids, with extreme nervous sensibility, about thirty-two hours.

3d. Comparative tranquillity, with a diminution of the hydrophobia and excessive susceptibility, twelve hours.

The dog was not known in the village, nor has it been ascertained from whence he came, or to whom he belonged. He was first seen at Kilburn on Sunday evening, May 7th, when he bit a man* and a boy†, and several dogs. He was afterwards fastened up for the night in Mr. Lovelock's stable, at "the Bells" public house, near the toll-gate, suffering himself to be tied without biting the person who secured him; but he broke loose during the night, and seized two horses in the adjoining stalls, one of which was attacked with hydrophobia on the 3d of June, twenty-four days after being bitten, and became extremely agitated and convulsed, kicking and tearing in such a violent manner as to endanger beating himself and the stable to pieces, until Mr. Lovelock caused him to be killed.

The dog was shot in the village soon after his attack upon the unfortunate gardener. The other dogs which were bitten were also destroyed. The man and the boy are still in good health. The boy was bitten in the upper arm, and the wounded parts were destroyed by caustic about two hours after the accident, by Mr. Chevalier, of South Audley-street.

The man was bitten in the arm likewise, but, owing to his thick clothing, only one tooth penetrated the flesh. Salt was immediately applied to the wound, rubbing it in until the part smarted. On the following day he went down to the sea-side, below Gravesend, where he continued four days, washing the part frequently with salt water, but did not put himself under the care of any medical attendant.

*Bloomsbury-square;* G. PINCKARD, M.D.

*July* 18, 1815.

---

* Thomas Chord, a shoemaker, ætat. 52, residing in the village of Kilburn.

† Richard Cole, æt. 11, a labourer's son, living also at Kilburn.

